# Medical Societies

**Published:** 1847-06

**Authors:** 


### ARTICLE II.

### MEDICAL SOCIETIES.

The ROCK RIVER MEDICAL SOCIETY which has, from time to time been noticed in our Journal, appears still to be in a prosperous condition, as will be seen by the following abstract from the proceedings of their last meeting furnished us for publication.

This is as it should be, and proves that the large body of intelligent physicians of the region included by the limits of the Society, properly appreciate the influence of such organizations in advancing the interests of the profession by diffusing information, exciting each other to emulation, causing investigations, and by promoting harmony and good fellowship amongst us.

Pursuant to adjournment, the Rock River Medical Society met, for the purpose of holding its annual meeting, in Rockford, Ill., May 18th, 1847.

The President, J. C. Goodhue, M. D., being in the chair, the meeting was duly organized by appointing Drs. Nash and Crosby, Vice Presidents pro tem., the regular Vice President being absent.

On motion, Drs. Payne, Ellis, Gregory, Spencer, Crosby, and Golliday were admitted members of the Society.

A paper was then read by Dr. S. G. Armor, on the *Pathology and Treatment of Periodical Fevers*, which led to a general expression of opinion on the subject by members of the Society. Interesting cases were also reported by Dr. Ames, detailing the character, progress and treatment of malignant fevers of a congestive form.

The following resolutions were offered by Dr. Lucius Clark, and unanimously adopted by the Society:

*Resolved*, That each member of this convention will use his exertions to elevate the standard of Medical Education in the west, by discouraging quackery in all its forms—by investigating assiduously the Medical Topography of his own region—by communicating the results of his observation to his professional brethren—And by sustaining, as far as is consistent with their merits, our western Medical Periodicals and western Medical Schools.

*Resolved*, That, as a *means* to elevate the standard of Medical Education, physicians should dissuade young men from entering the profession whose preparatory education is deficient.

*Resolved*, That as facilities for teaching the several branches of Medical Education are much greater at public institutions than they possibly can be under the instruction of private preceptors, students should be discouraged from taking upon themselves the responsibility of the healing art, until they shall have graduated in their profession, at some respectable school.

The Society then proceeded to ballot for officers for the ensuing year, which resulted in the election of the following gentlemen, to-wit:

*President*—J. B. Nash, M. D.

*First Vice President*—A. Clark, M. D.

*Second Vice President*—C. Martin, M. D.

*Secretary*—R. S. Moloney, M. D.

*Treasurer*—S. G. Armor, M. D.

*Censors*—A. W. Bentworth, M. D.

J. C. Goodhue, M. D.

The chair appointed W. W. Welch, M. D., to read a paper before the Society at its next annual meeting.

On motion,

*Voted*, That the Society hold an evening session at the Court House, at 7 o'clock, P. M., at which time a lecture was delivered on the subject of *Homœpathy* by Dr. Armor. Interesting remarks, relating more particularly to the *practice* founded on the theory of this school of doctors, were made by Dr. Goodhue.

On motion,

*Voted*, That the next annual meeting of this Society be held at Dixon, Lee county, Illinois, on the third Tuesday in May next, and that a semi-annual meeting be held in Beloit, Wiskonsin Territory, on the third Tuesday in November.

S. G. ARMOR,
*Secretary Rock River Medical Society.*

The UNION MEDICAL SOCIETY of northern Indiana, held its third annual meeting in Goshen, on the second Tuesday in May last.

Papers were read as follows: by Dr. Henkle on Quackery, by Dr. Willard on Erysipelas, and by Dr. Parks on Pneumonia; which last excited a spirited discussion.

Dr. Ellis, the President, then delivered an address which we are sorry our limits forbid us from laying before our readers.

These Societies have now stood out their probation, and may be considered as permanently established.

We hope our brethren in other districts will follow the example set them by the Rock River and Northern Indiana physicians in organizing themselves into bodies for mutual benefit. What more refreshing to the careworn practitioner, than occasionally thus to meet with friends with whom he has a common interest, who can appreciate his toils for afflicted man; who think in unison with him on topics that mutually engage their attention; who have the same trials and difficulties to contend with, and who also join in his consolations. Certainly such scenes, with such men, could but be productive of a "flow of soul," and of those

"Greenest spots in Memory's waste."

---

ARTICLE III.

